# Supplementary material for: Synthesis and Characterization of Andrographolide Derivatives as Regulators of βAPP Processing in Human Cells
Source: Molecules. 2021 Dec 17;26(24):7660. doi: 10.3390/molecules26247660 (PMC8707718; doi:10.3390/molecules26247660)

Synthesis and Characterization of Andrographolide Derivatives as Regulators of βAPP Processing in Human Cells

Arpita Dey[a,1], Ran Chen[b,1], Kun Dai[b], Subhamita Maitra[a], Feng Li[b], Jean-Francois Hernandez[c], Guo-Chun Zhou[b,*] and Bruno Vincent[a,d,*]

[a] Institute of Molecular Biosciences, Mahidol University, Nakhon Pathom, 73170 Thailand

[b] School of Pharmaceutical Sciences, Nanjing Tech University, Nanjing, Jiangsu 211816, China

[c] Institut des Biomolécules Max Mousseron, UMR5247 CNRS/Université de Montpellier/ENSCM, Faculté de Pharmacie, 34093 Montpellier Cedex 5, France

[d] Centre National de la Recherche Scientifique, 2 rue Michel Ange, 75016 Paris, France

Running title: Andrographolide derivatives as βAPP processing modulators

[*] Corresponding authors: bruno.vin@mahidol.ac.th (B. Vincent), gczhou@njtech.edu.cn (G.-C. Zhou)

[1] These authors contributed equally to this work

Supplementary materials

Contents

NMR spectra for Scheme 1 ---page s3-s5

NMR spectra for Scheme 2---page s6-s23

(14α)-(Quinolyl-2'-methyl-5',7'-dichloro-8'-oxy)-3,19-acetonylidene andrographolide (**11**):

^{1}H NMR of **11**:

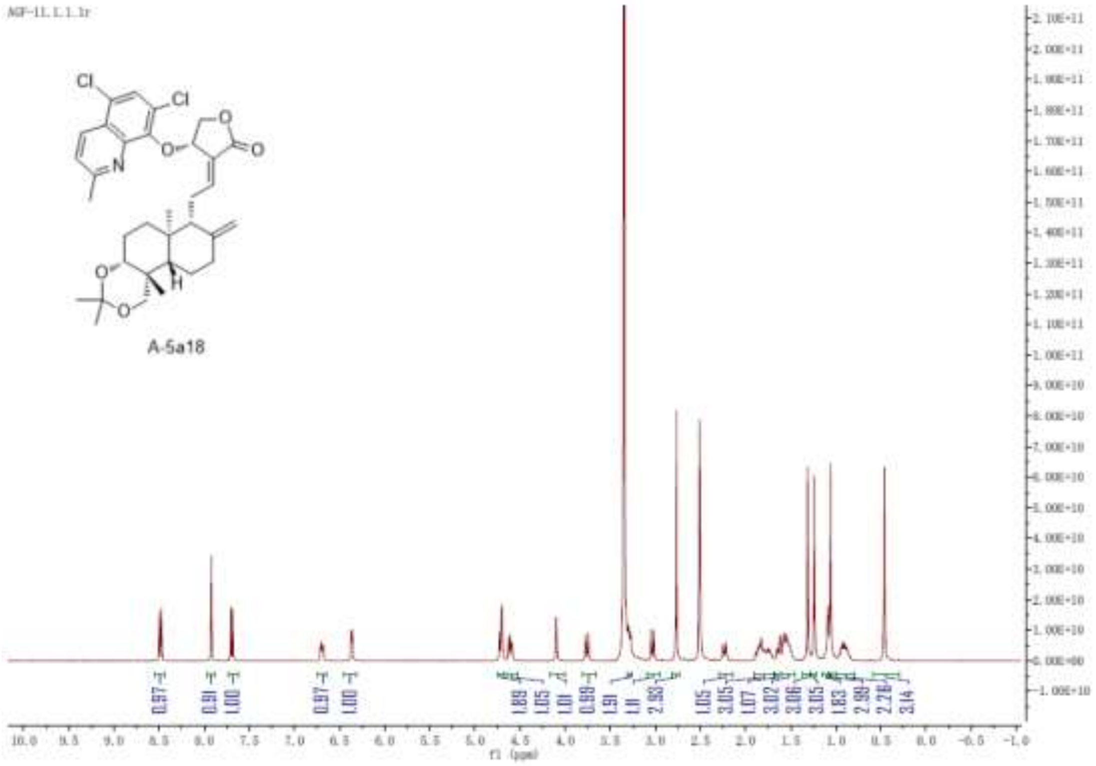

^{13}C NMR of **11**:

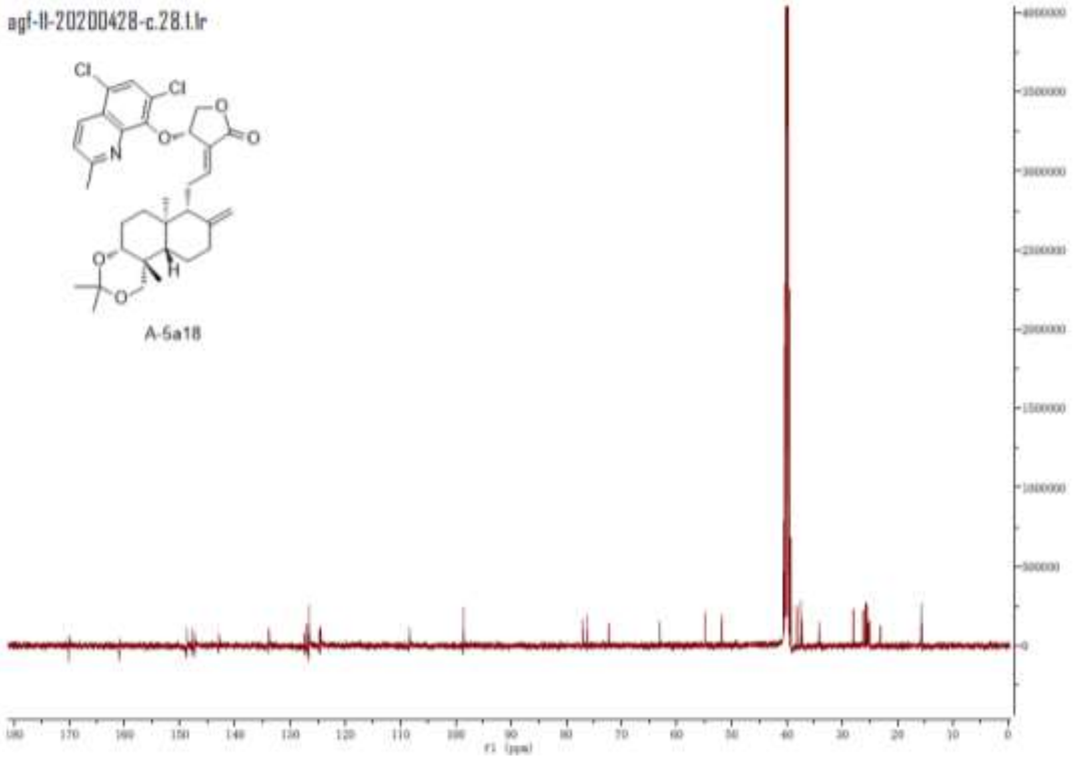

(14α)-(Quinolyl-2'-methyl-5',7'-dichloro-8'-oxy) andrographolide (**17**):

^{1}H NMR of **17**:

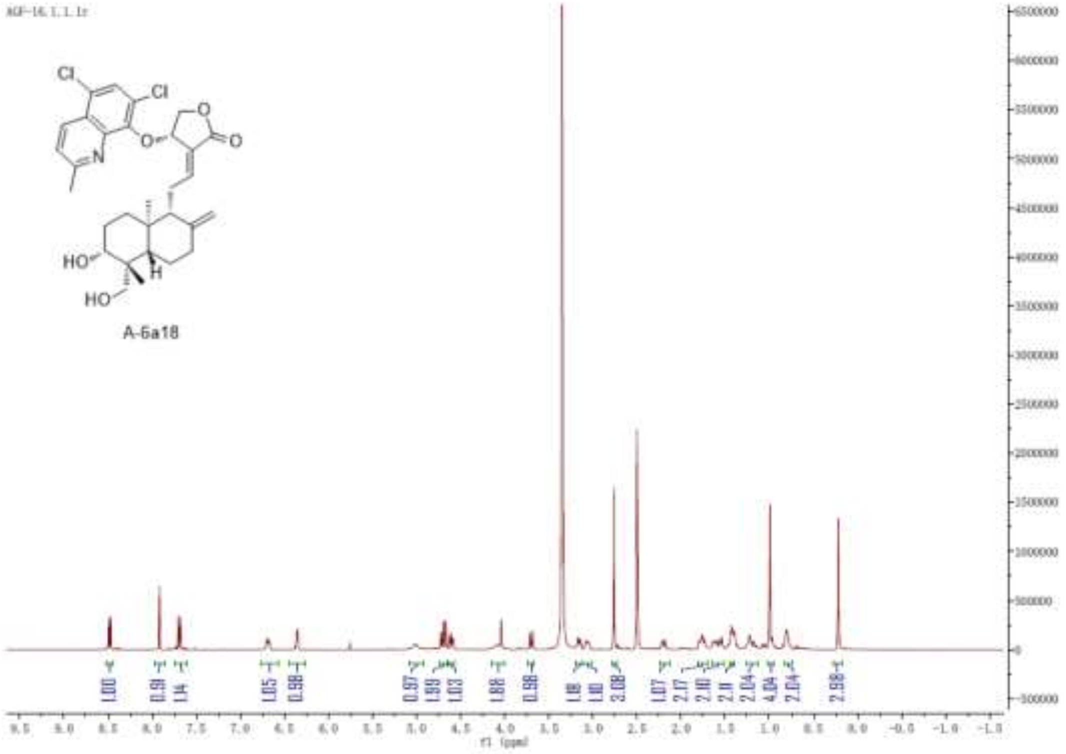

^{13}C NMR of **17**:

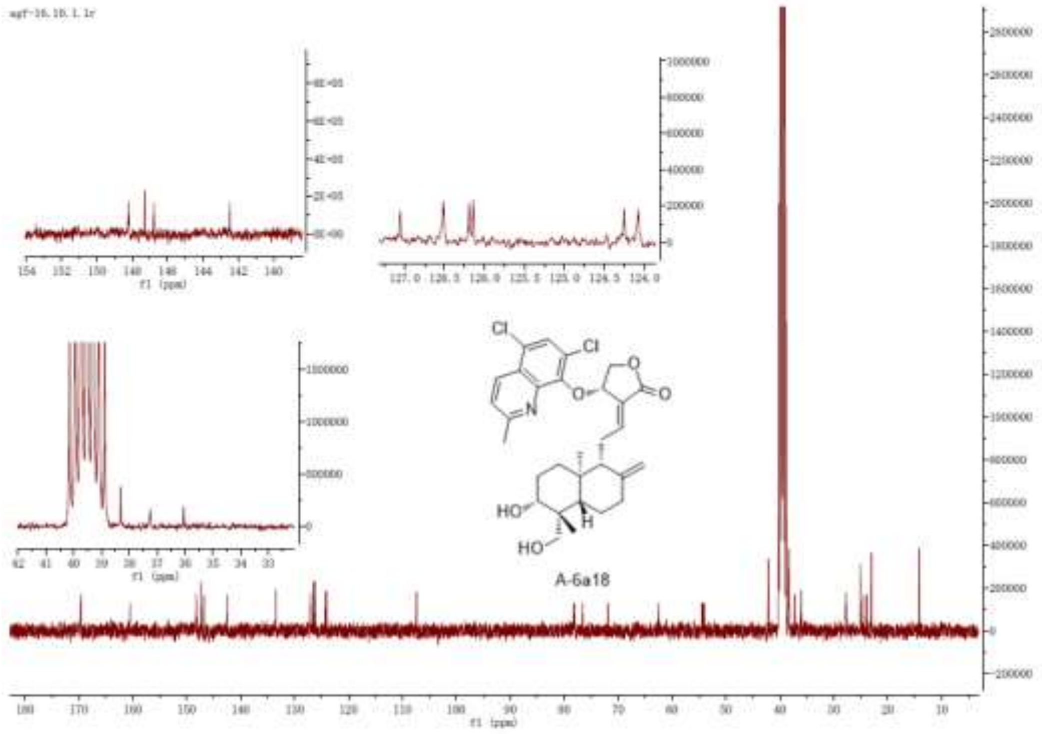

(14β)-(Quinolyl-2'-methyl-5',7'-dichloro-8'-oxy) andrographolide (**18**):

^{1}H NMR of **18**:

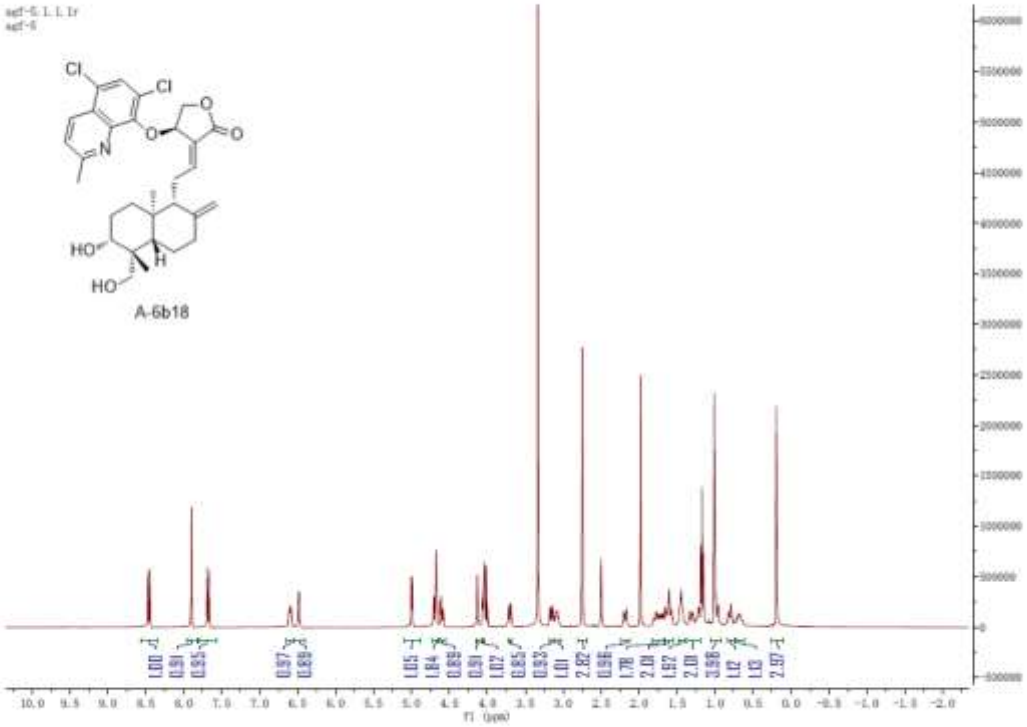

^{13}C NMR of **18**:

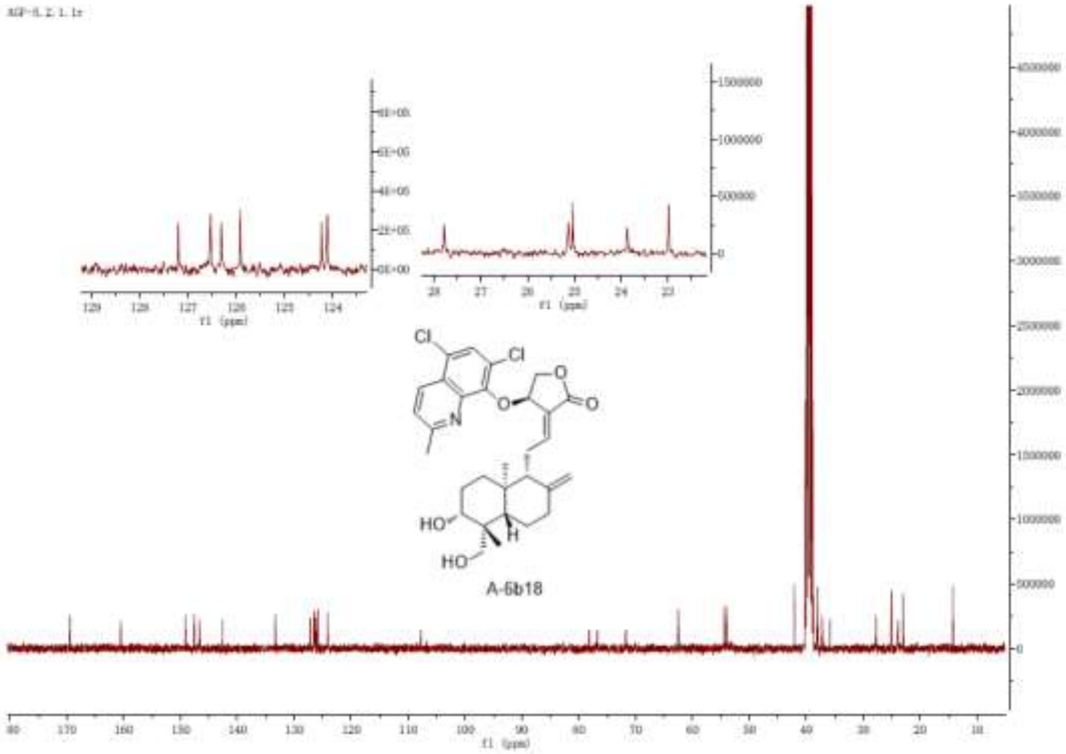

(14α)-(4’-nitrobenzoyl) -3,19-isopropylideneoxy-andrographolide (**19**).

^{1}H NMR of **19**:

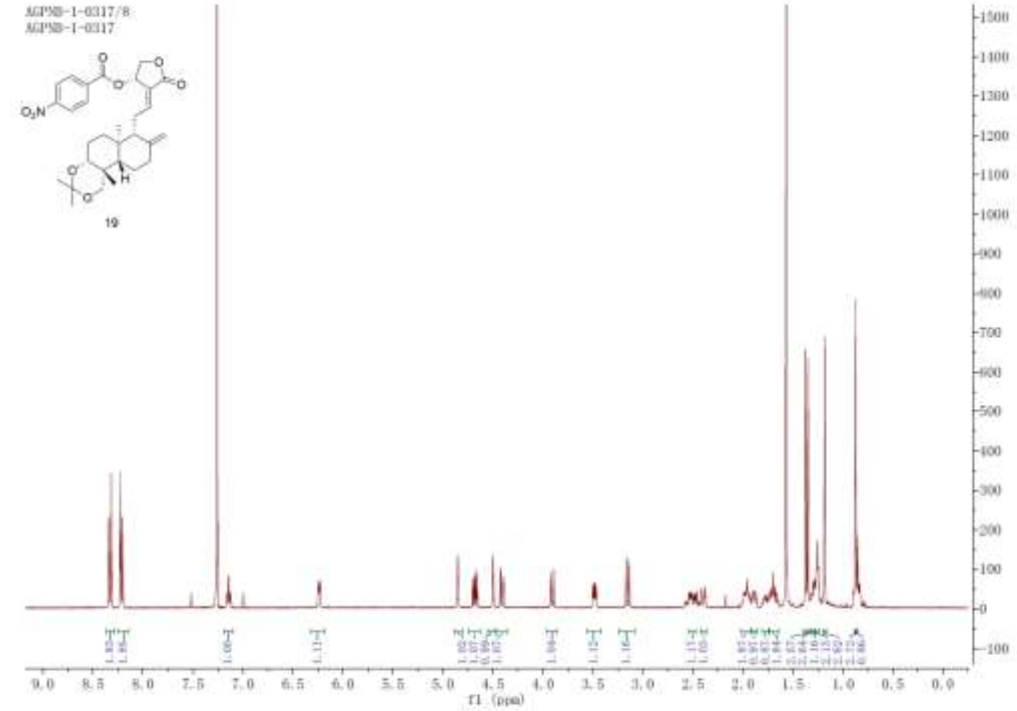

(14β)-(4’-nitrobenzoyl) -3,19-isopropylideneoxy-andrographolide (**20**).

^{1}H NMR of **20**:

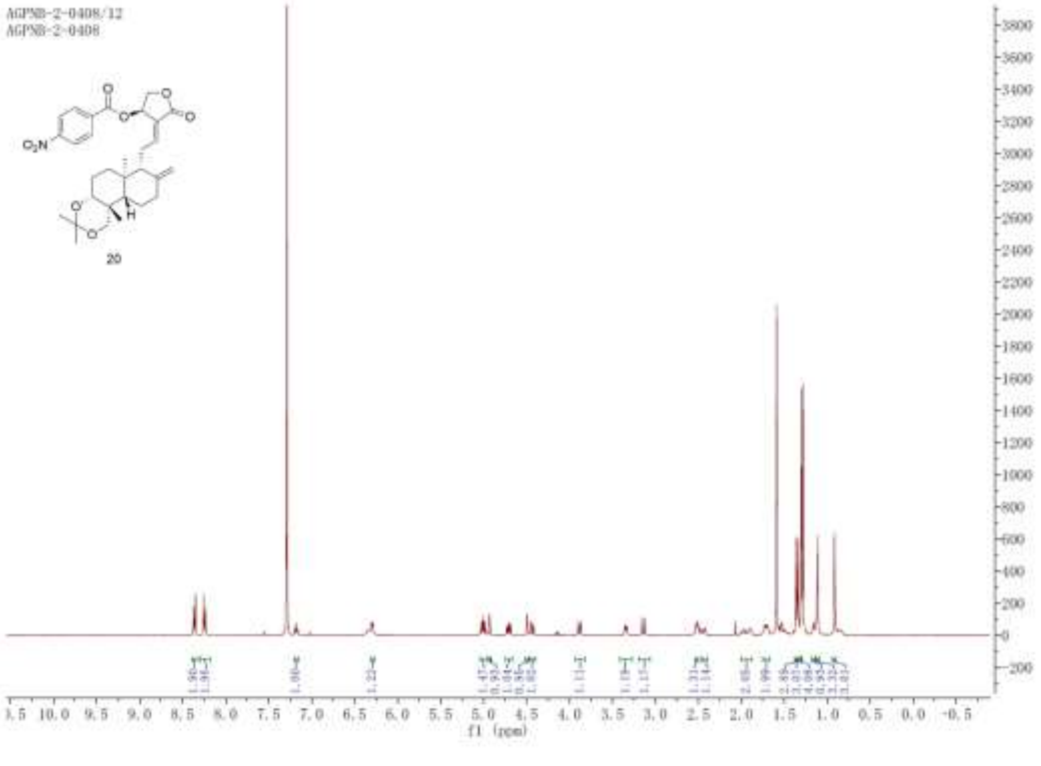

(14α)-(4’-nitrobenzoyl) -9-dehydro-17-hydro-andrographolide (**21**).

^{1}H NMR of **21**:

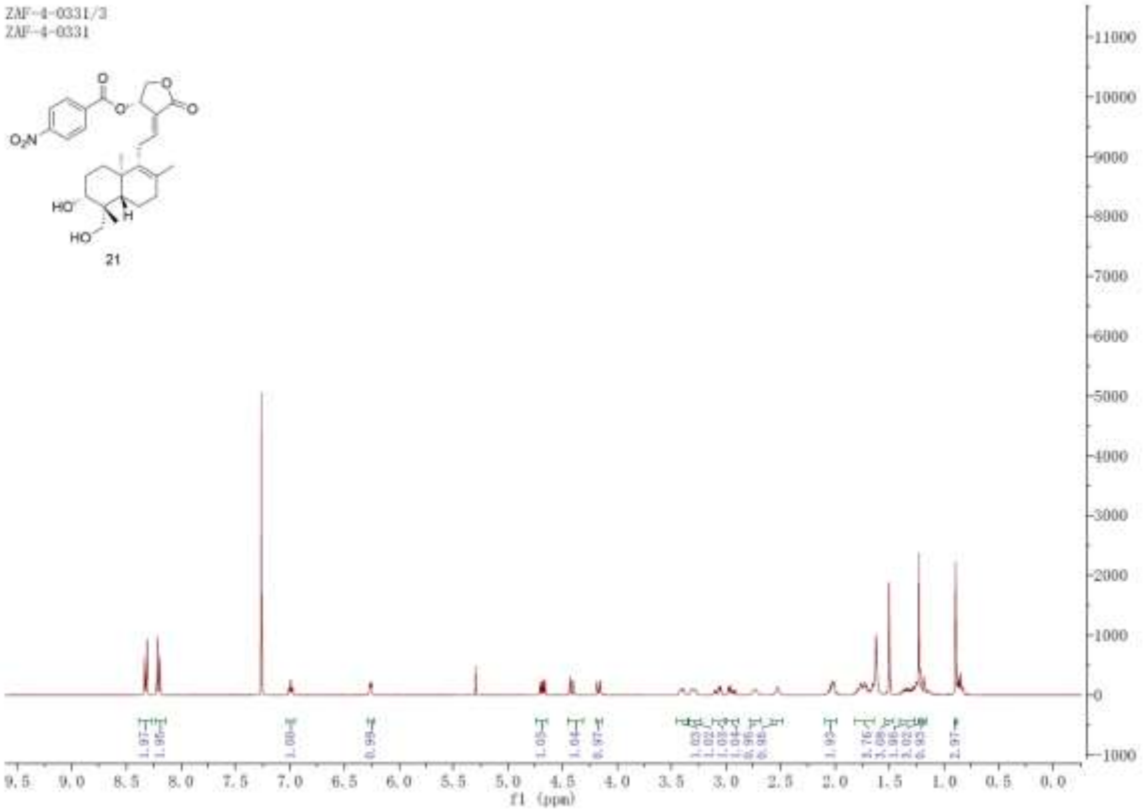

^{13}C NMR of **21**:

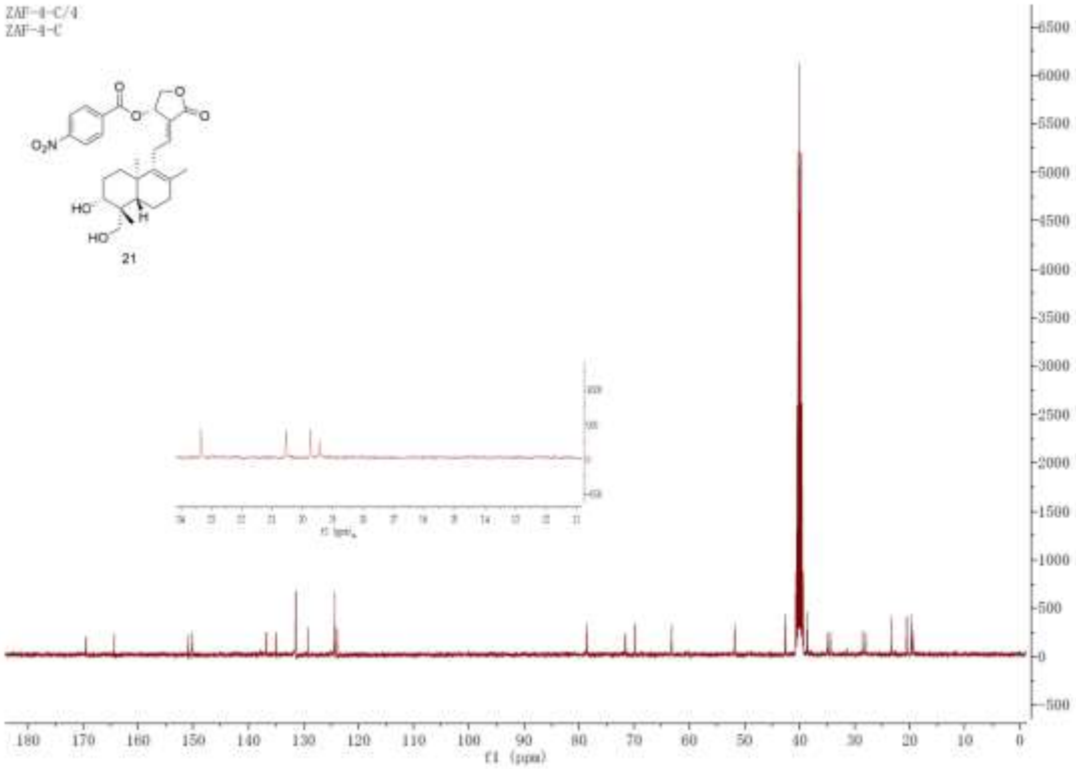

(14β)-(4'-nitrobenzoyl) -9-dehydro-17-hydro andrographolide (**22**).

^{1}H NMR of **22**:

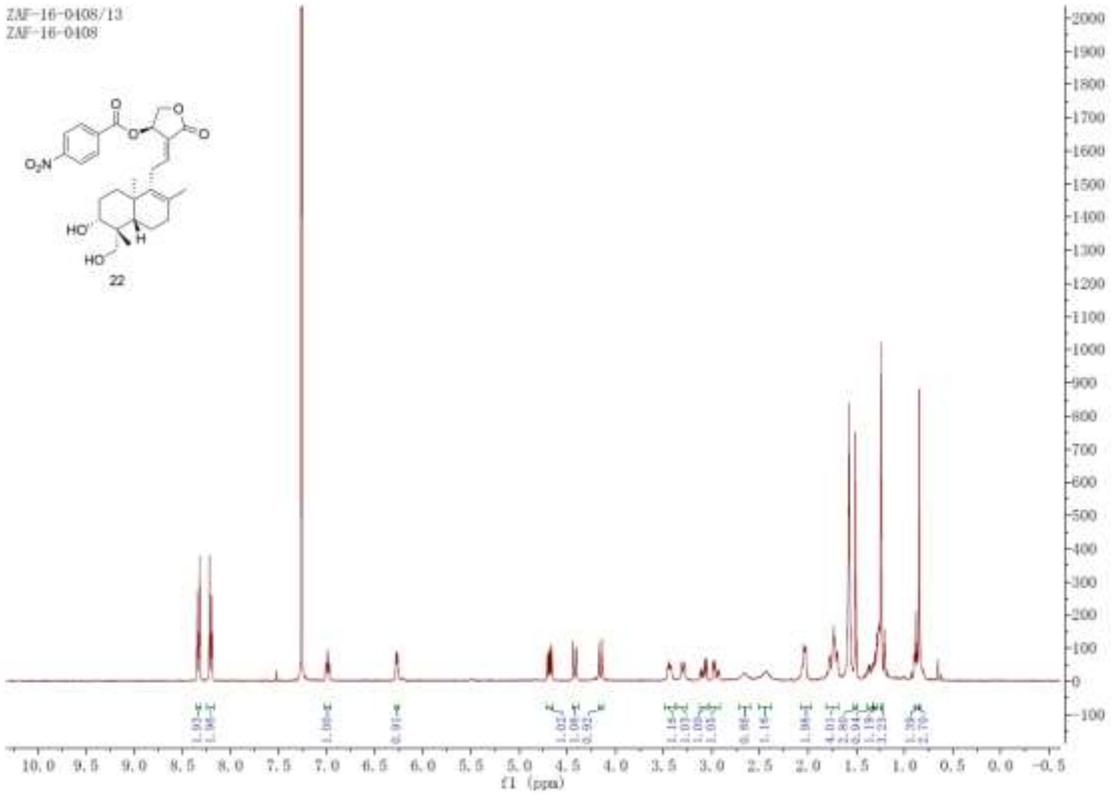

^{13}C NMR of **22**:

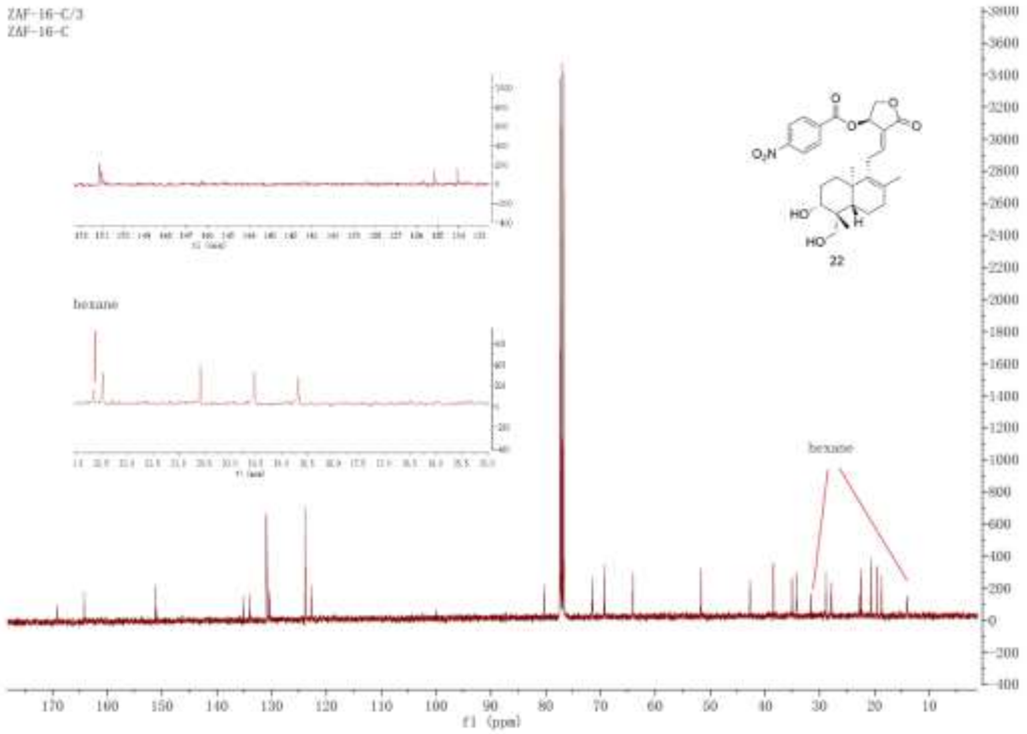

(14α)-(4’-nitrobenzoyl)-9-dehydro-17-hydro-3,19-isopropylideneoxy andrographolide (**23**).

^{1}H NMR of **23**:

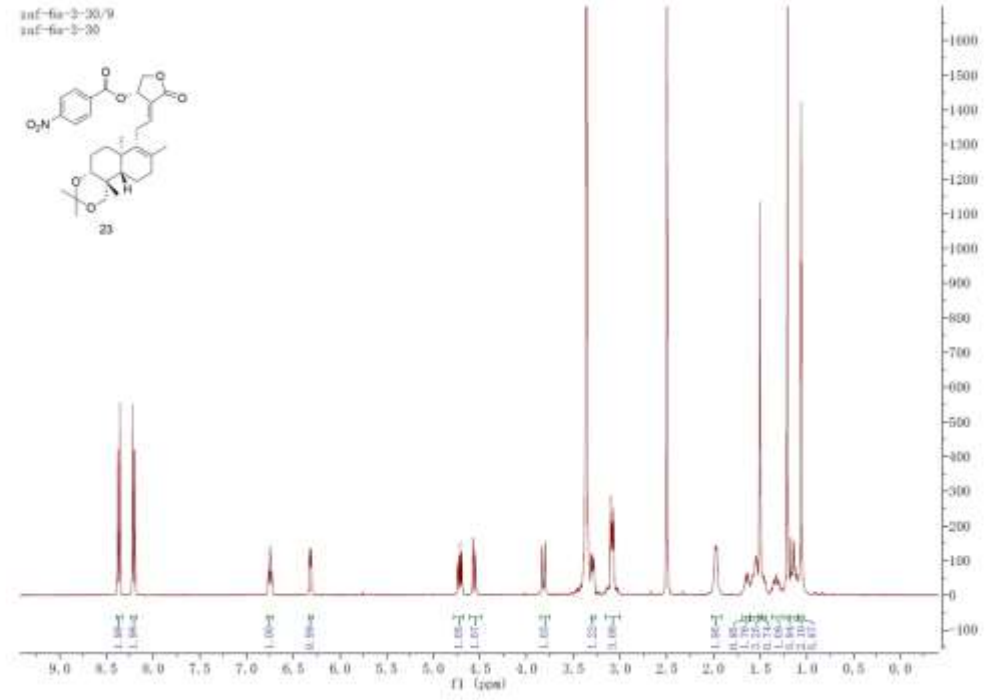

(14β)-(4’-nitrobenzoyl)-9-dehydro-17-hydro-3,19-isopropylideneoxy andrographolide (**24**).

^{1}H NMR of **24**:

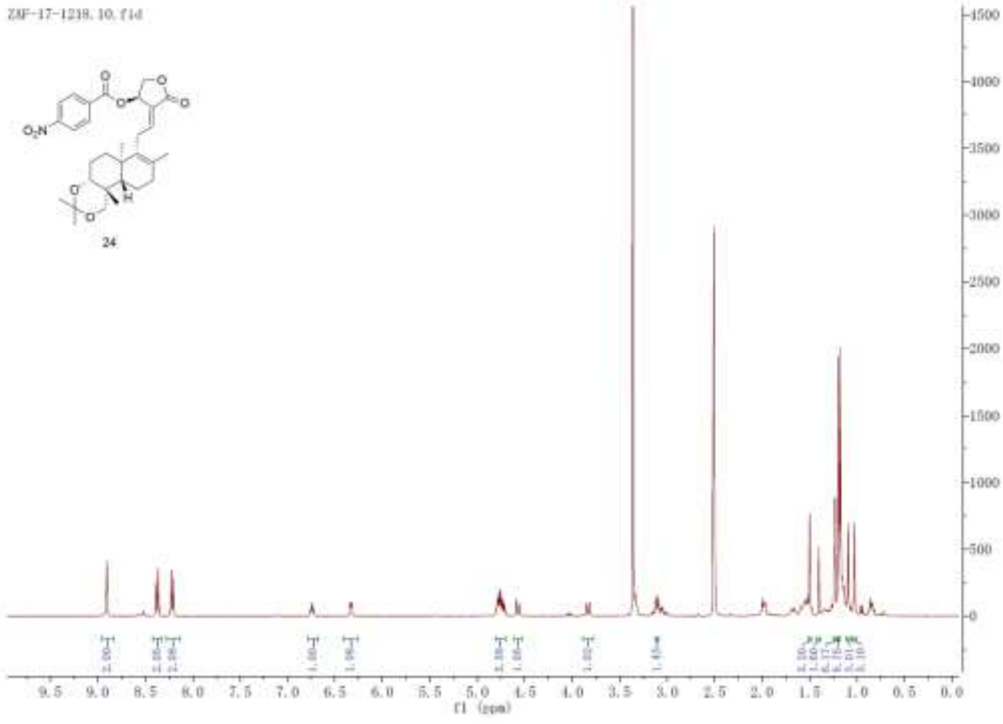

(14α)-9-dehydro-17-hydro-3,19-isopropylideneoxy andrographolide (**25**).

^{1}H NMR of **25**:

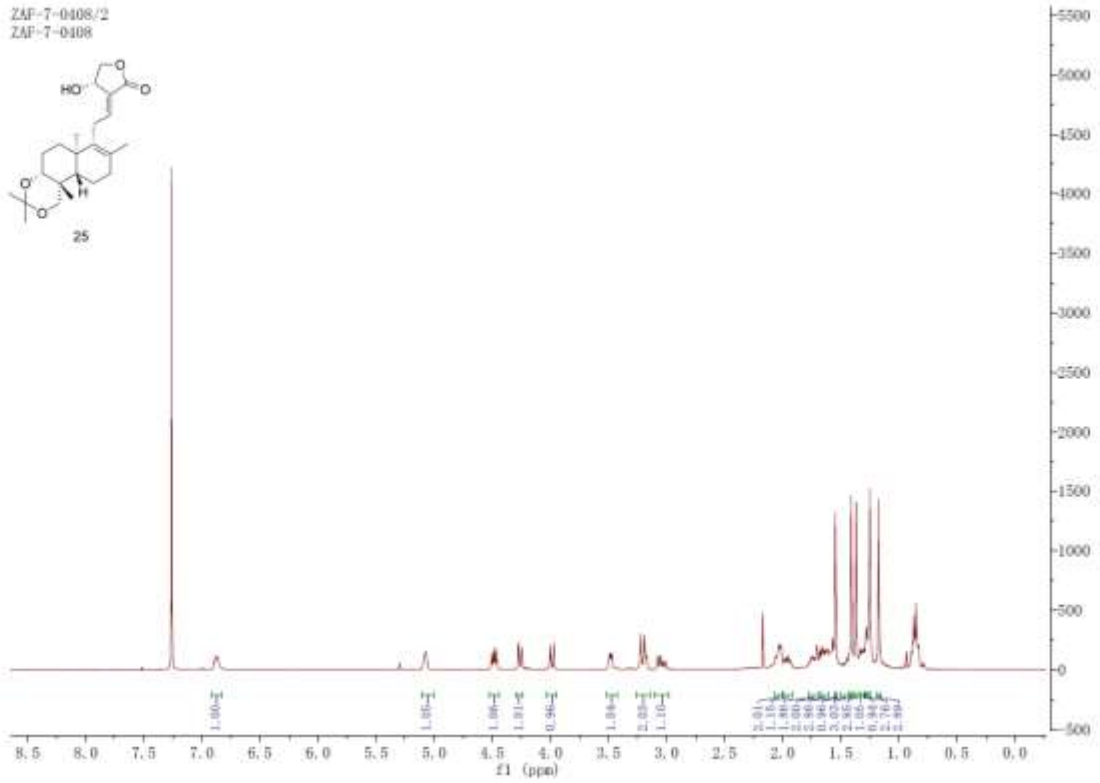

^{13}C NMR of **25**:

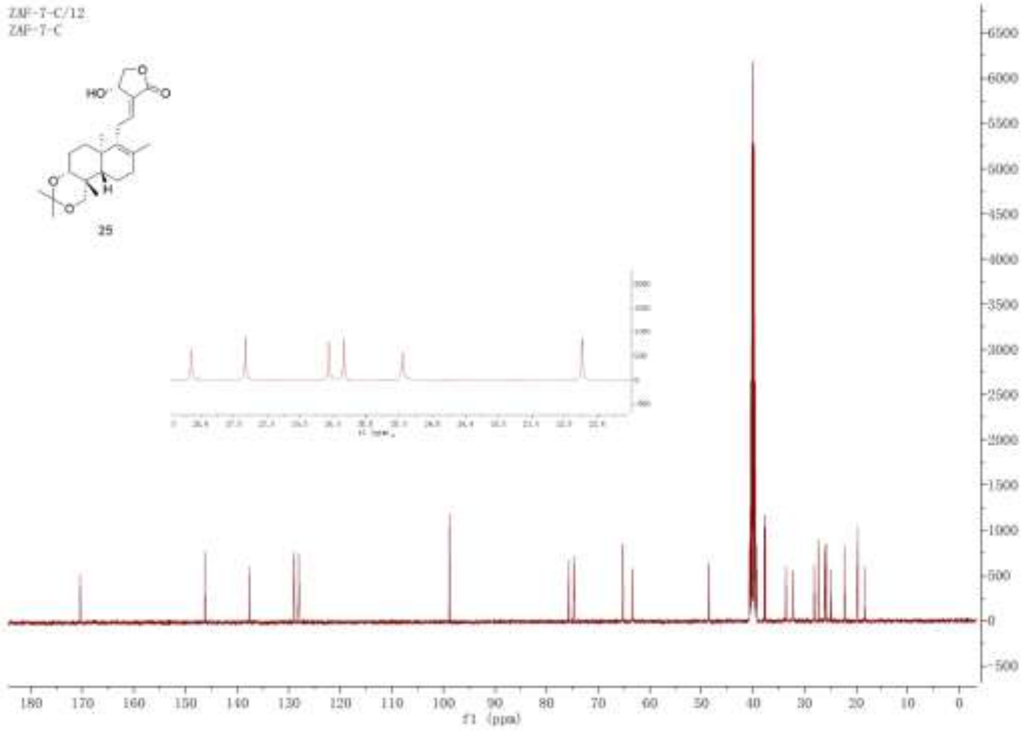

(14β) -9-dehydro-17-hydro - 3,19-isopropylideneoxy-andrographolide (**26**).

^{1}H NMR of **26**:

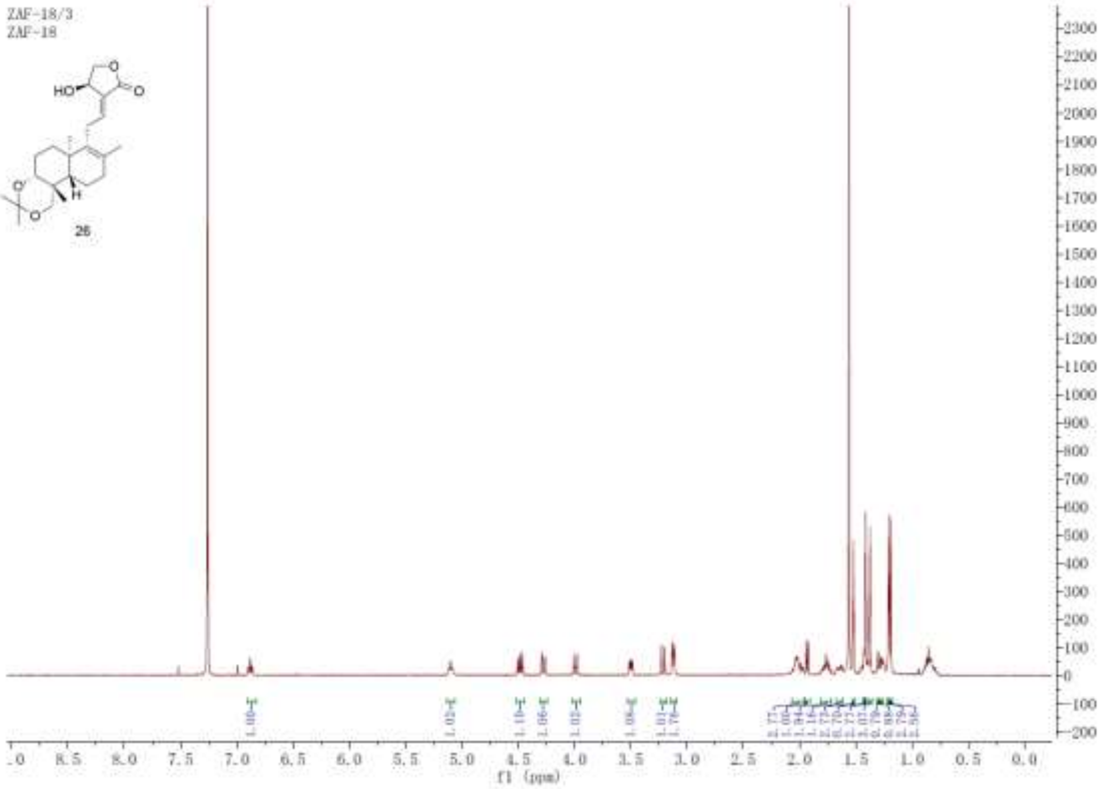

^{13}C NMR of **26**:

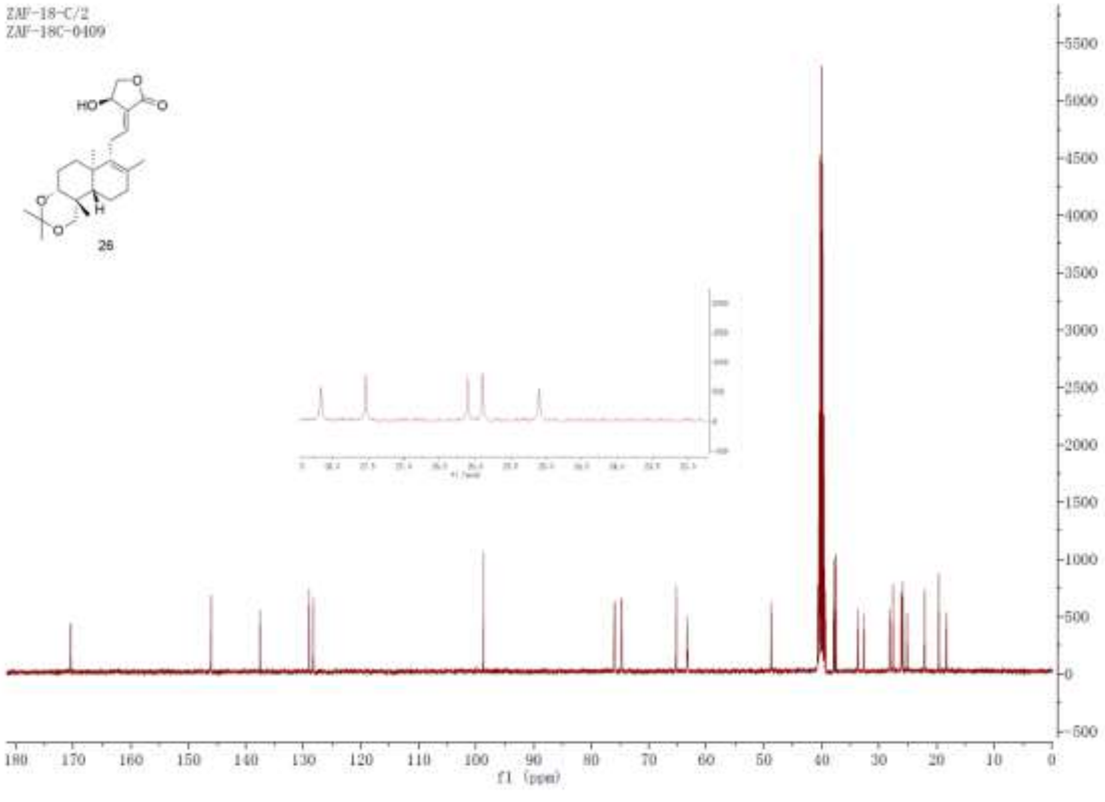

(14α)-(2’-Methyl-8’-quinolinoxy)-9-dehydro-17-hydro-3,19-isopropylideneoxy andrographolide (**27**):

^{1}H NMR of **27**：

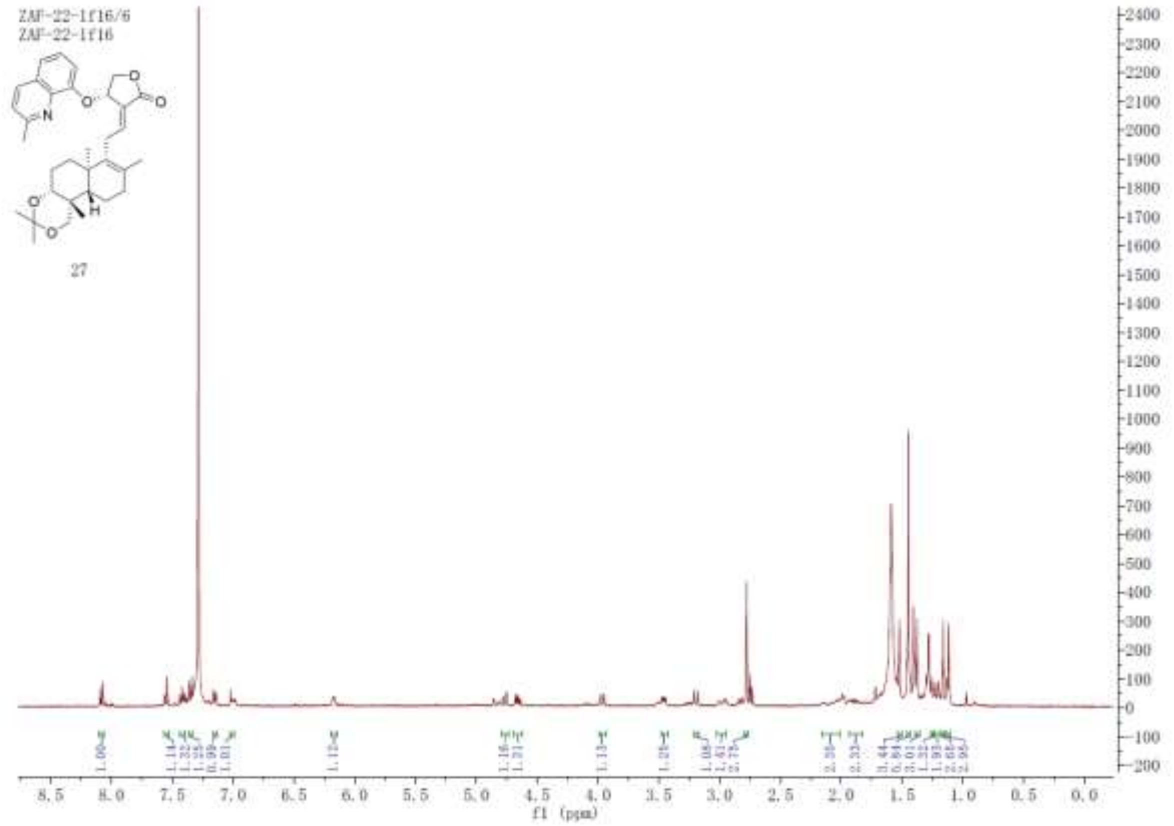

(14β)-(2’-Methyl-8’-quinolinoxy)-9-dehydro-17-hydro-3,19-isopropylideneoxy andrographolide (**28**):

^{1}H NMR of **28**:

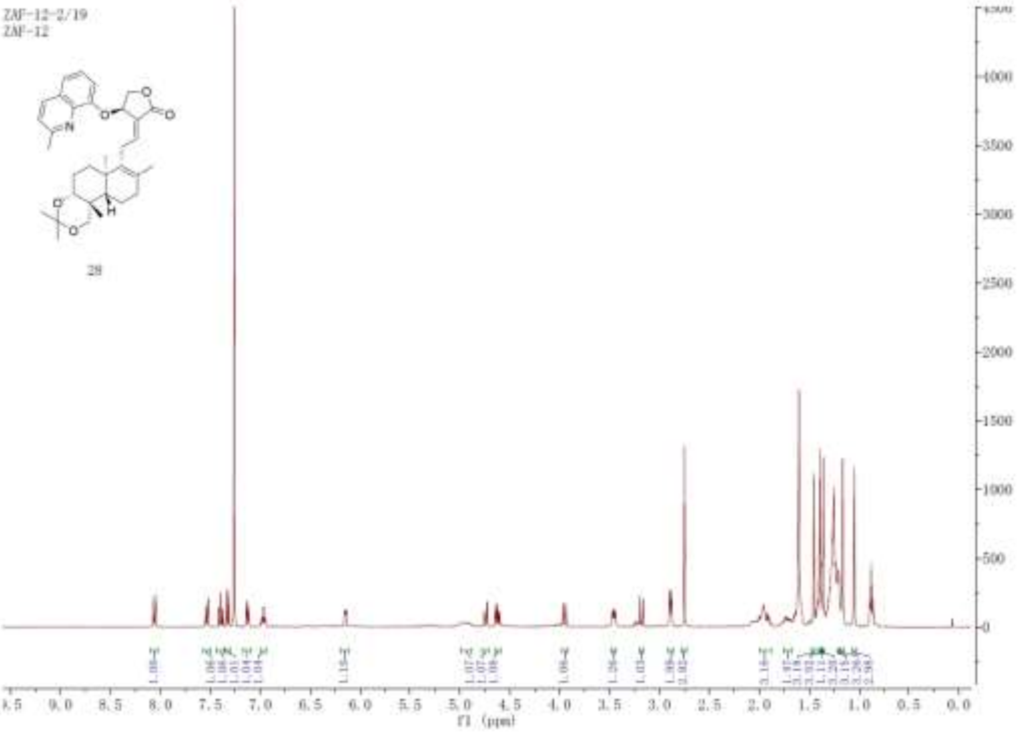

^{13}C NMR of **28**:

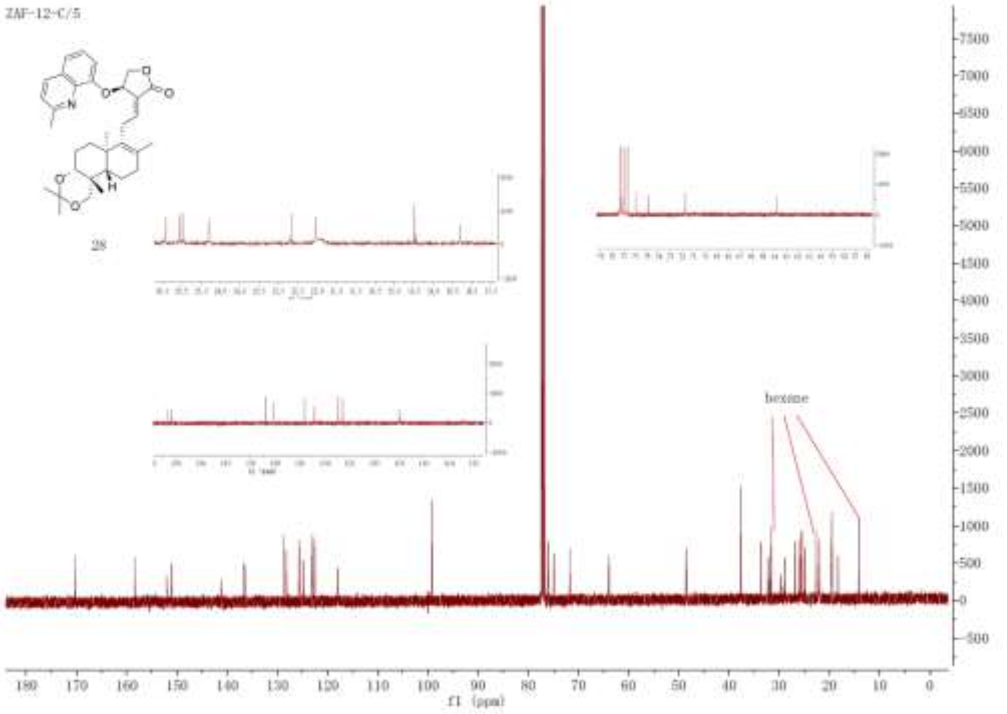

(14α)-(5’,7’-Dichloro-8’-quinolinoxy) -9-dehydro-17-hydro -3,19-isopropylideneoxy-andrographolide (29):

^{1}H NMR of **29**:

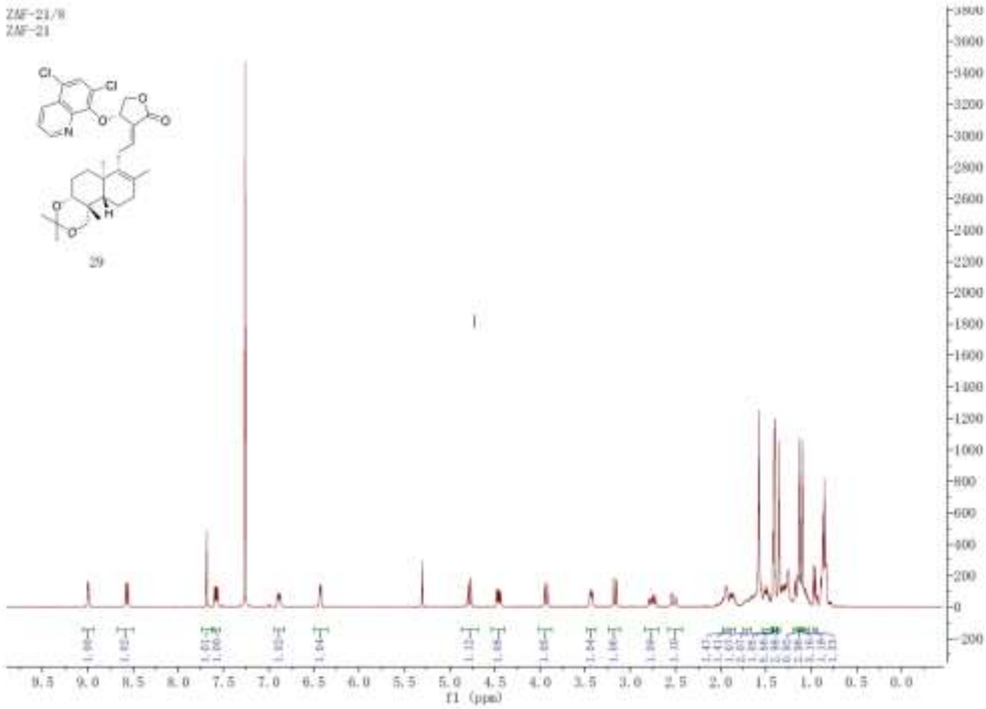

^{13}C NMR of **29**:

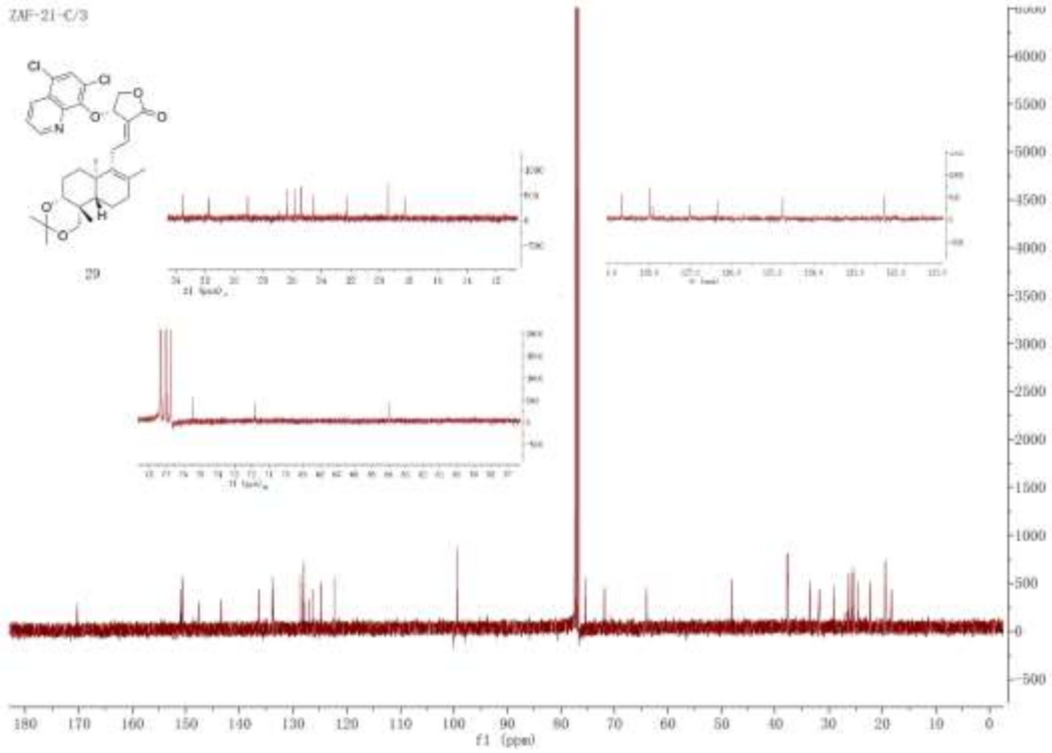

(14β)-(2’-Methyl-5’,7’-dichloro-8’-quinolinoxy)-3,19-isopropylideneoxy andrographolide (**30**):

^{1}H NMR of **30**:

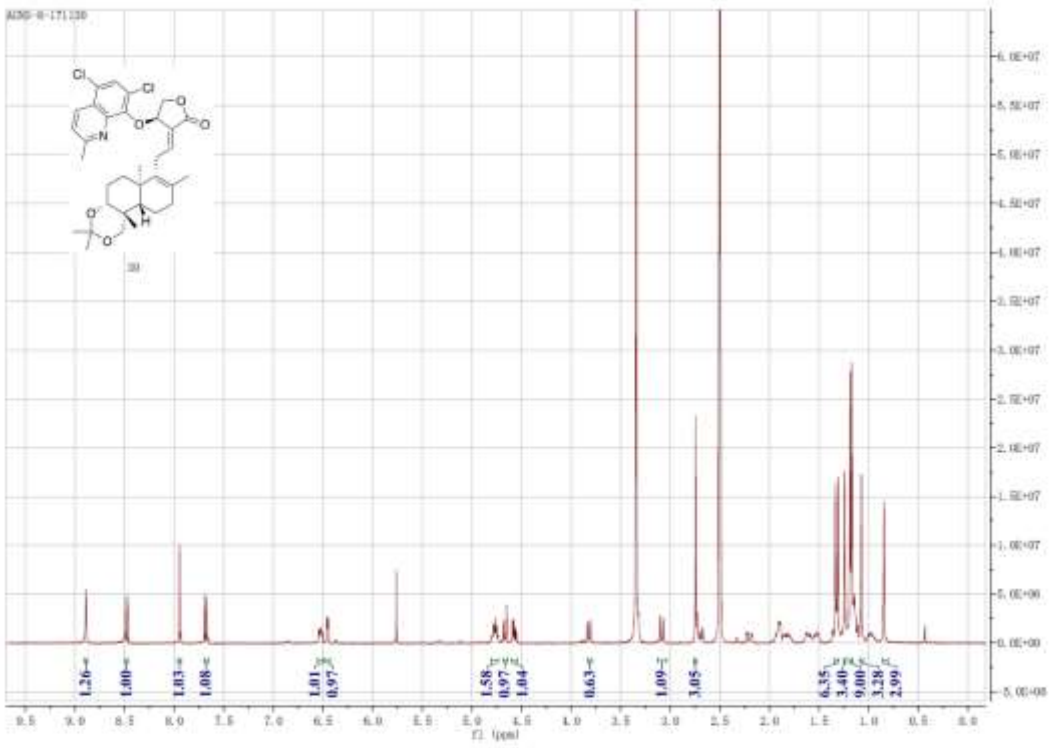

^{13}C NMR of **30**:

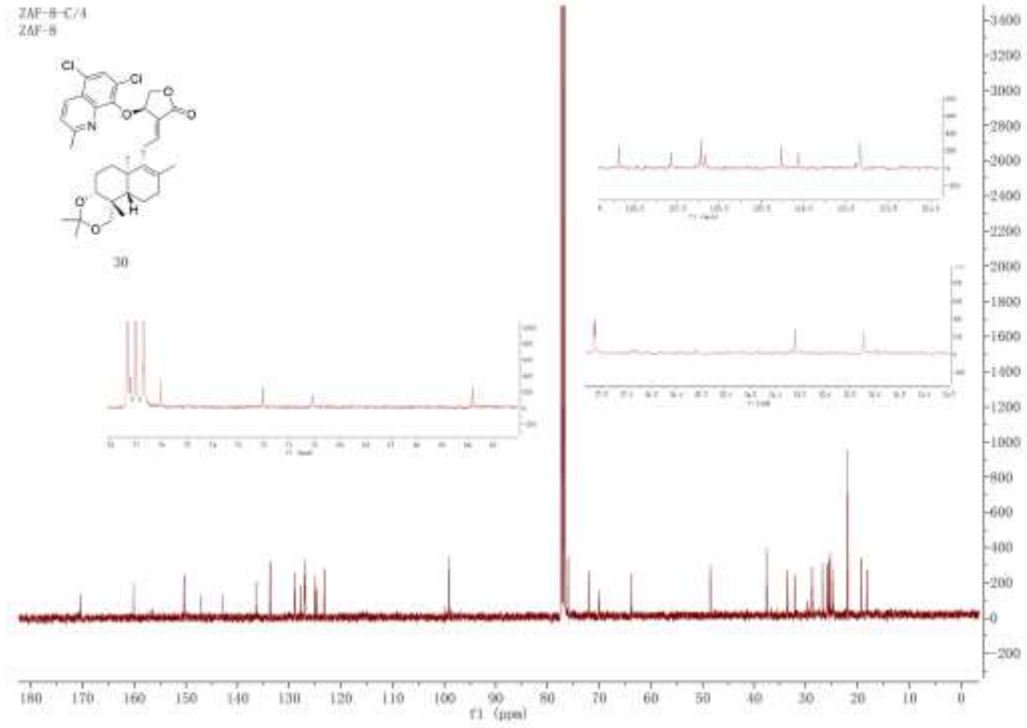

(14α)-(2’-Methyl-5’,7’-Dichloro-8’-quinolinoxy)-9-dehydro-17-hydro -3,19-isopropylideneoxy andrographolide (**31**):

^{1}H NMR of **31**:

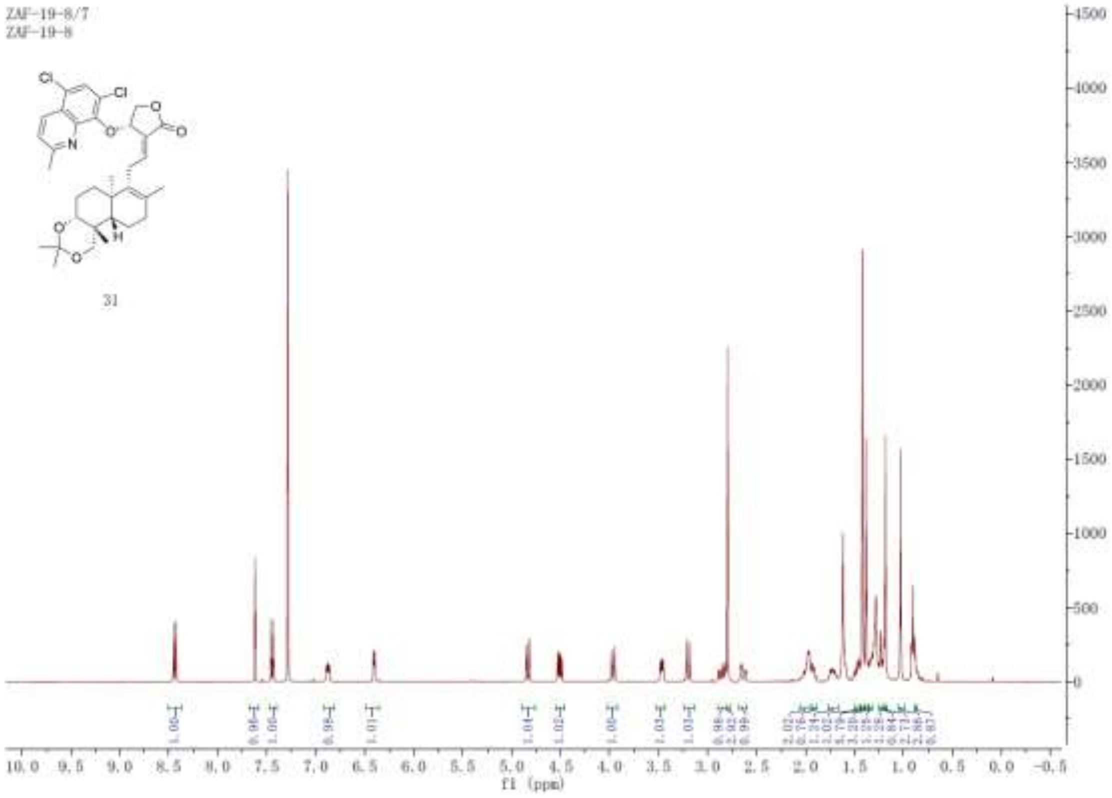

^{13}C NMR of **31**:

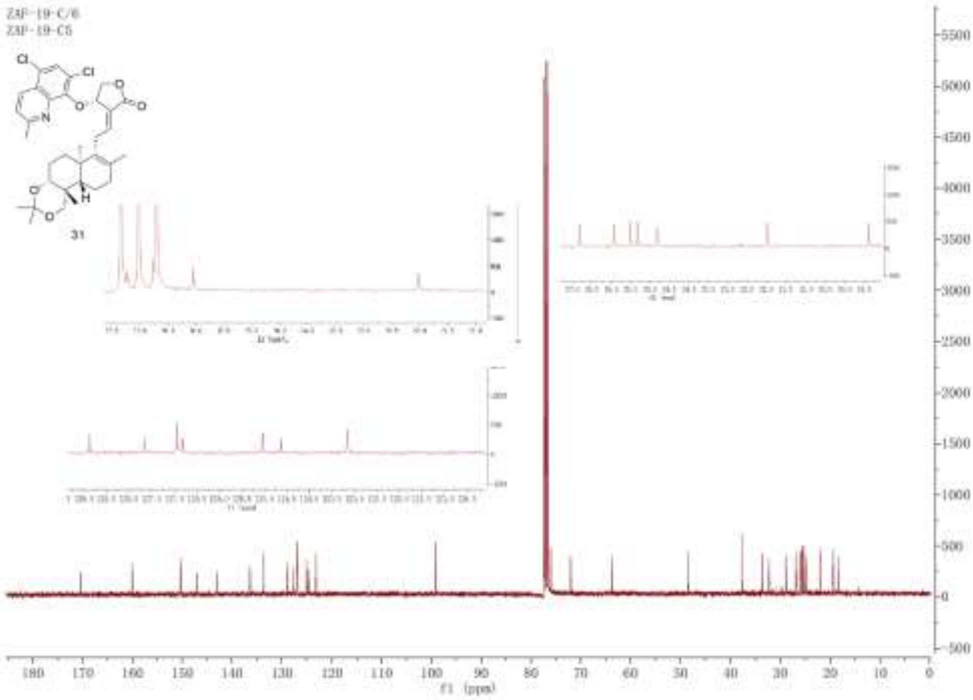

(14β)-(5’,7’-Dichloro-8’-quinolinoxy)-9-dehydro-17-hydro-3,19-isopropylideneoxy andrographolide (**32**) :

^{1}H NMR of **32**:

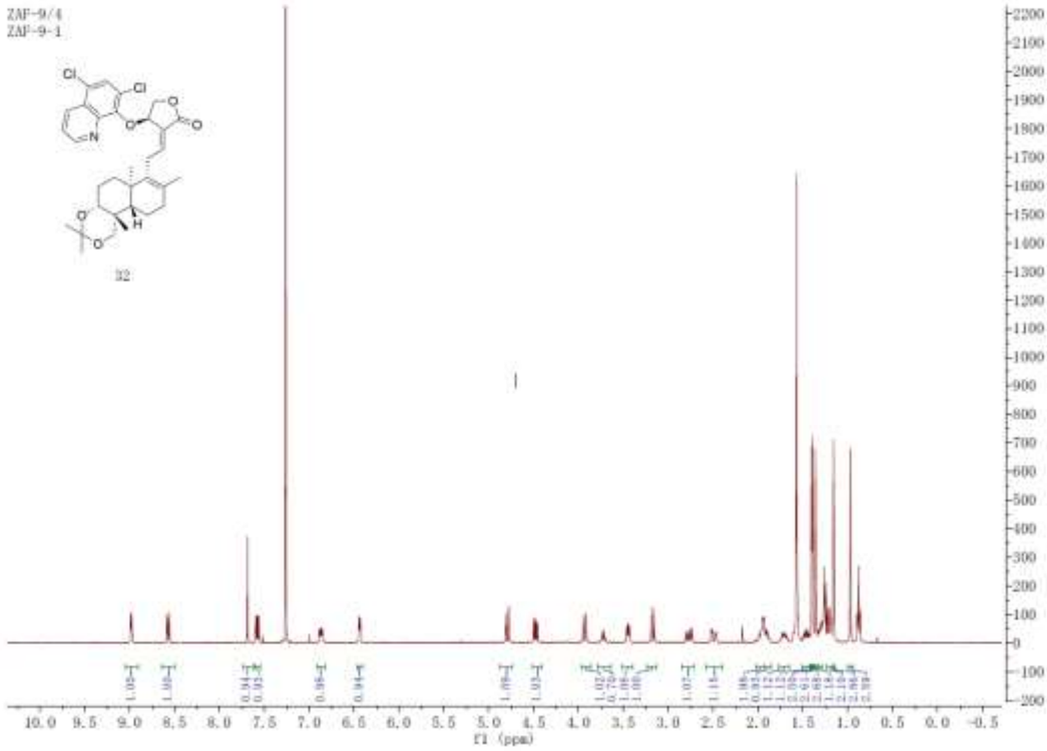

^{13}C NMR of **32**:

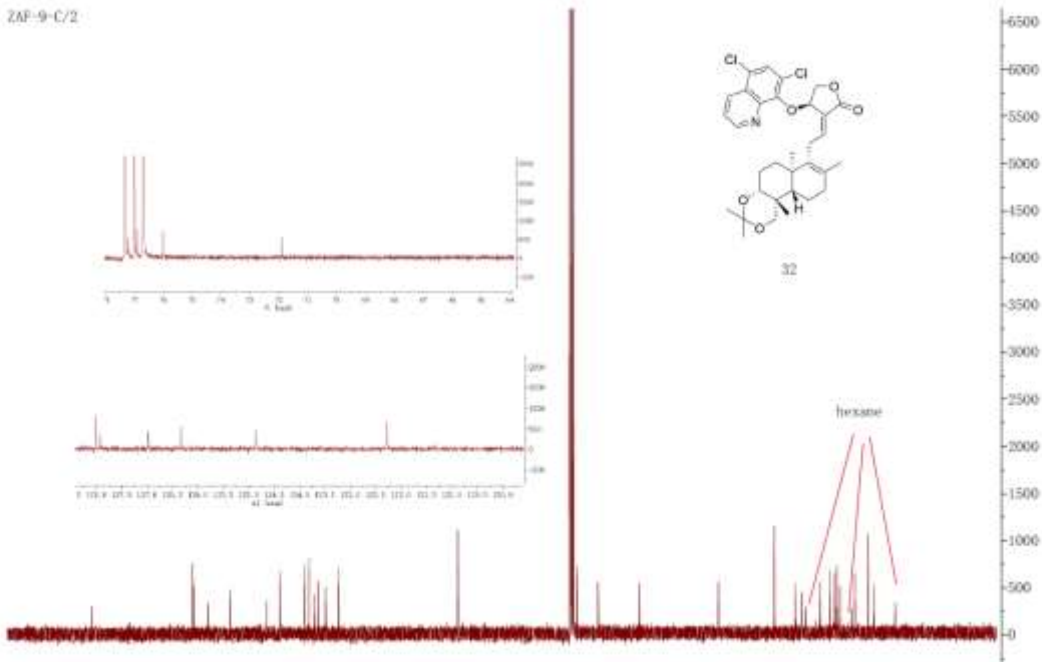

(14α)-(2’-Methyl-8’-quinolinoxy)-9-dehydro-17-hydro andrographolide (**33**):

^{1}H NMR of **33**:

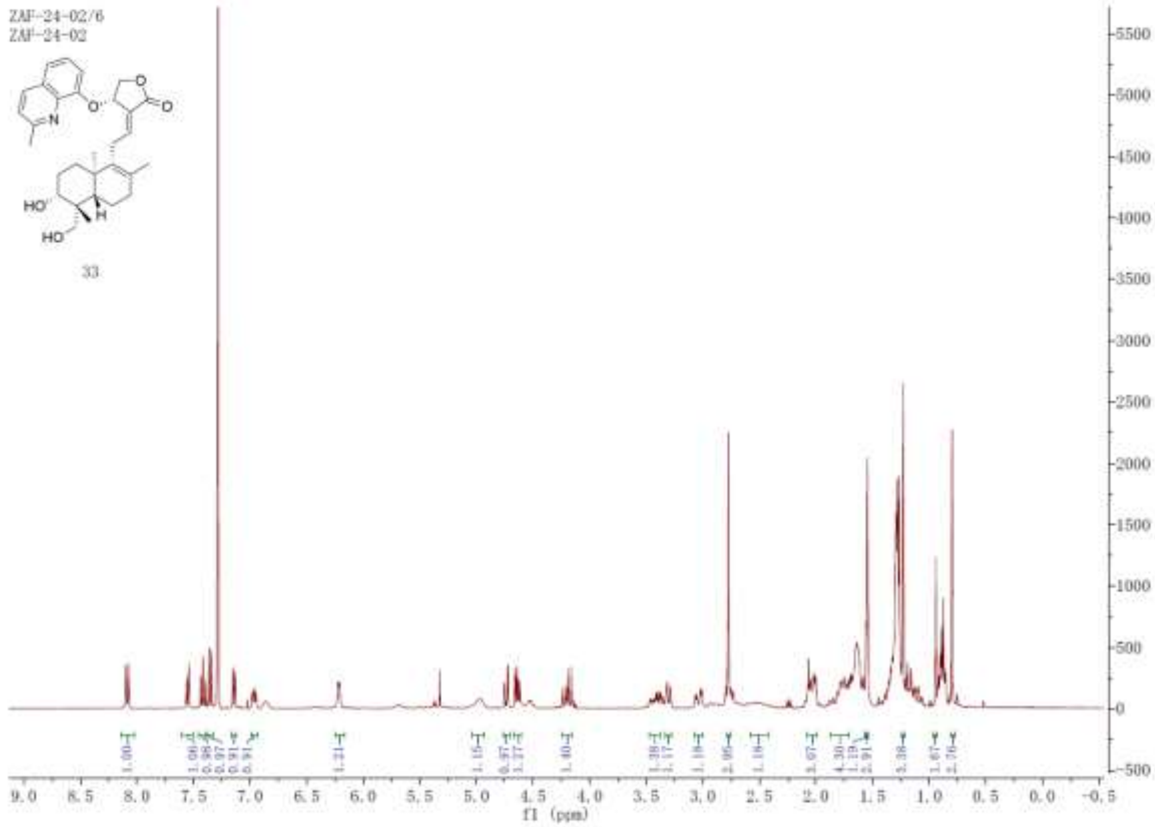

(14β)-(2'-Methyl-8'-quinolinoxy)-9-dehydro-17-hydro andrographolide (**34**):

^{1}H NMR of **34**:

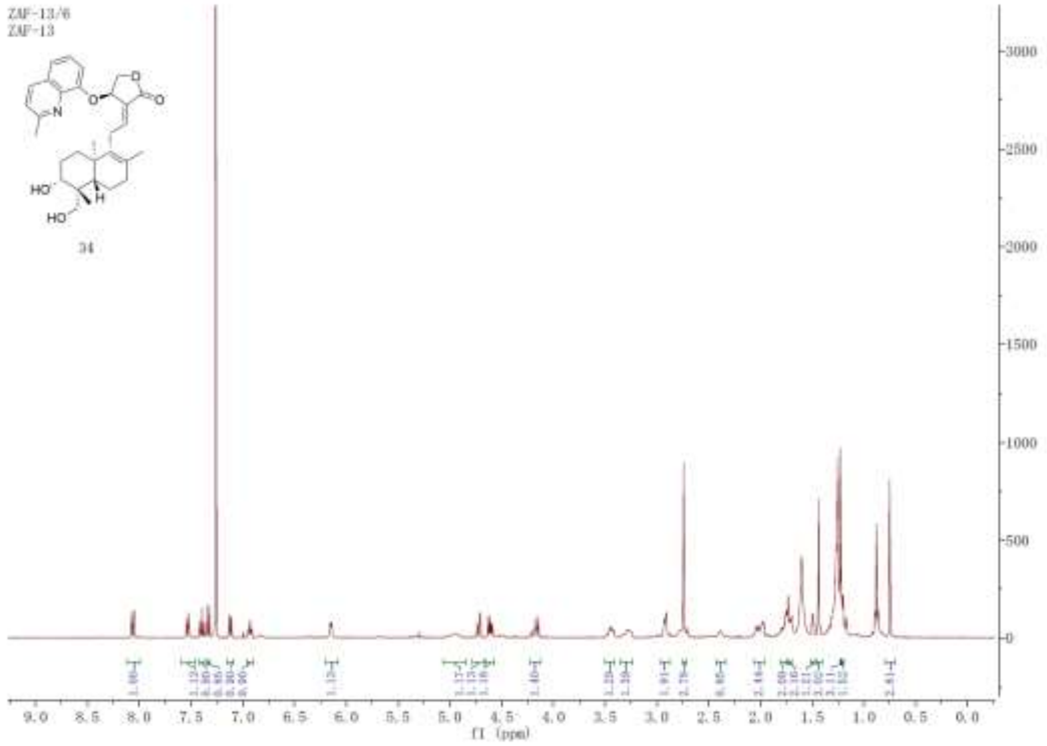

^{13}C NMR of **34**:

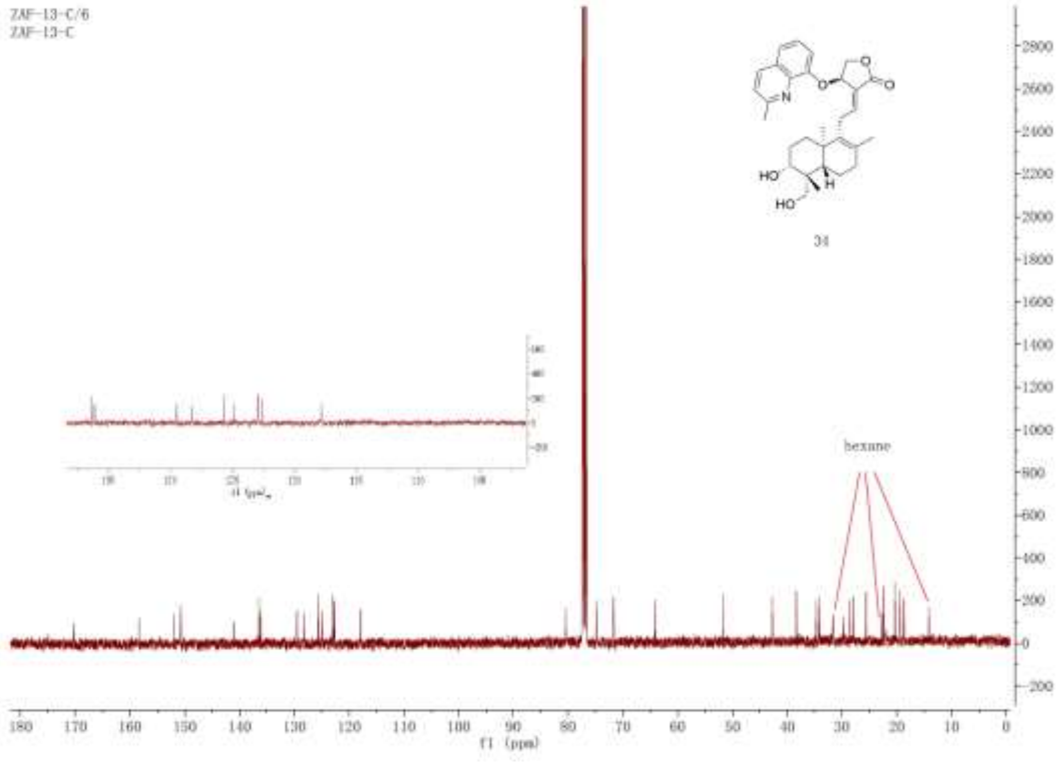

(14α)-(5’,7’-Dichloro-8’-quinolinoxy)-9-dehydro-17-hydro andrographolide (**35**):

^{1}H NMR of **35**:

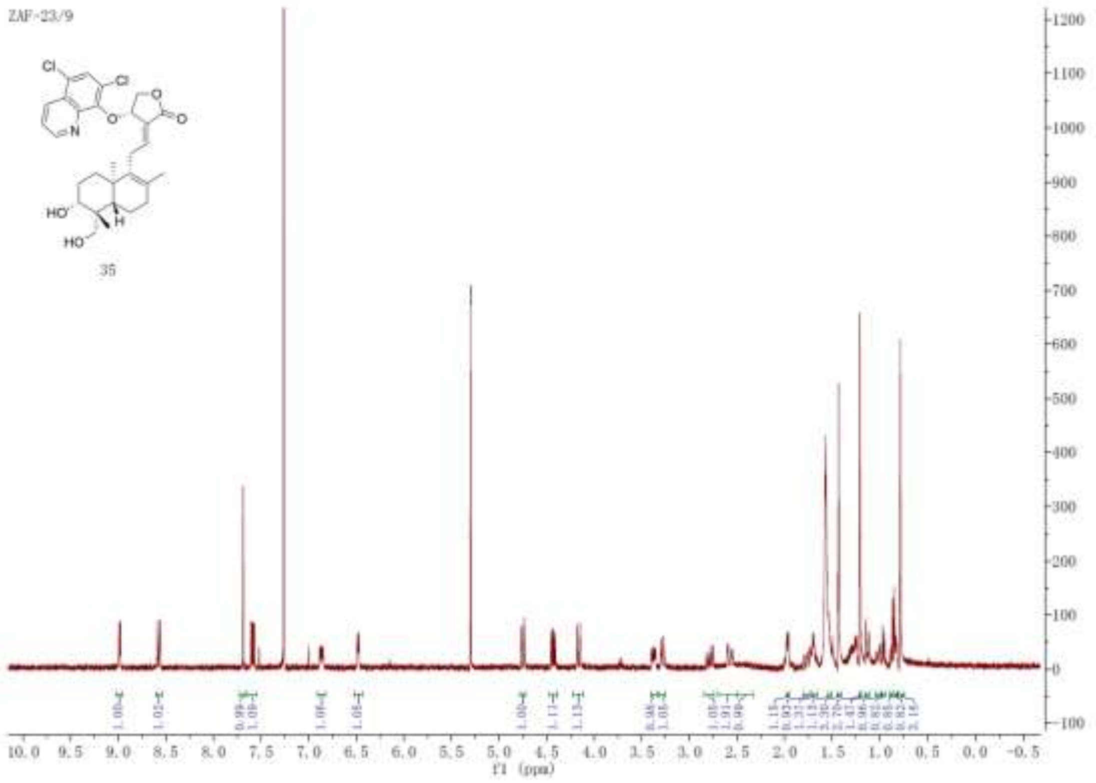

^{13}C NMR of **35**:

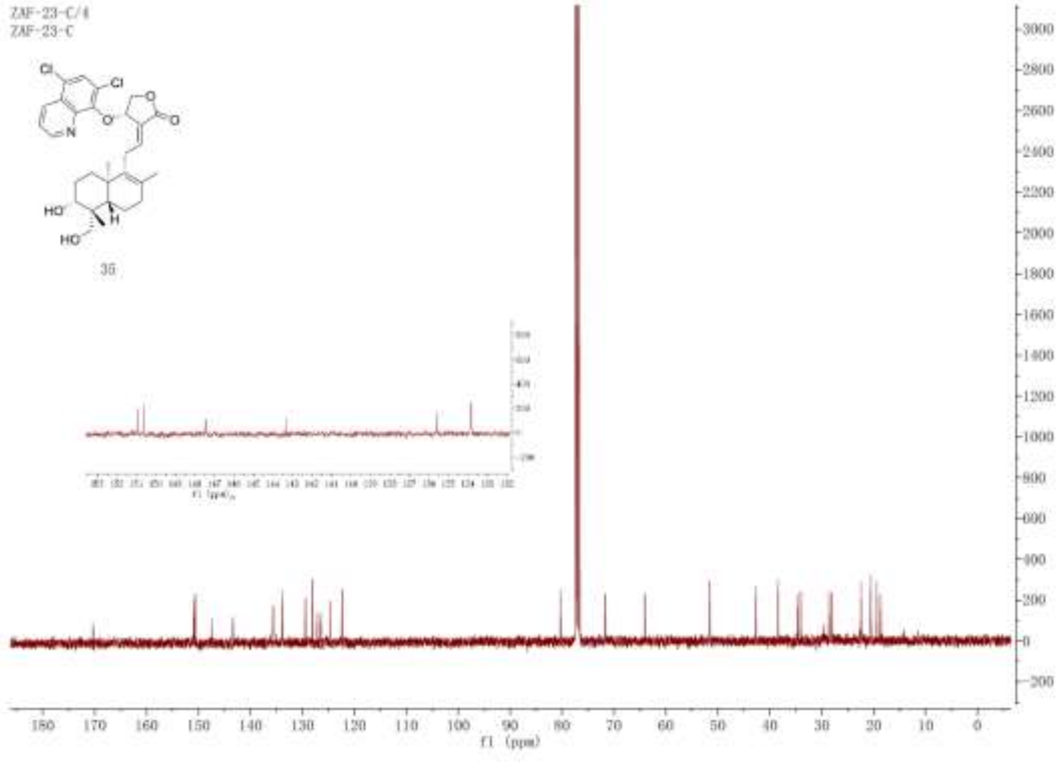

(14β)-(5’,7’-Dichloro-8’-quinolinoxy)-9-dehydro-17-hydro andrographolide (**36**):

^{1}H NMR of **36**:

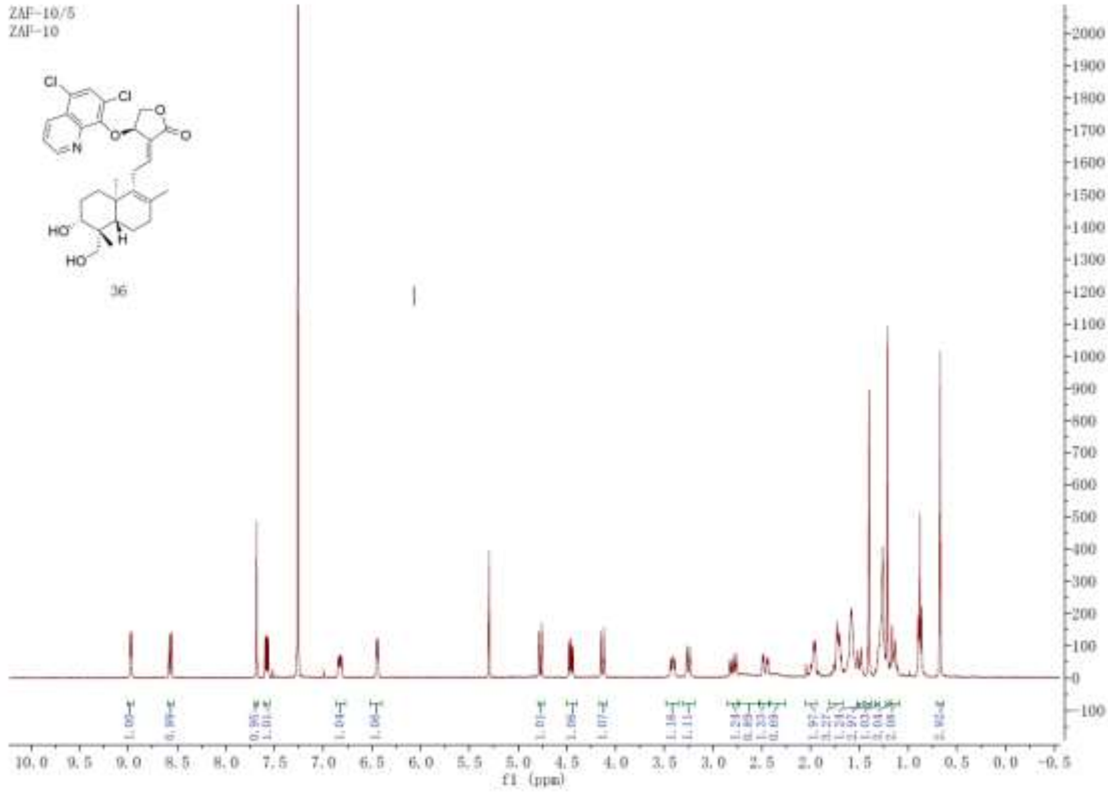

^{13}C NMR of **36**:

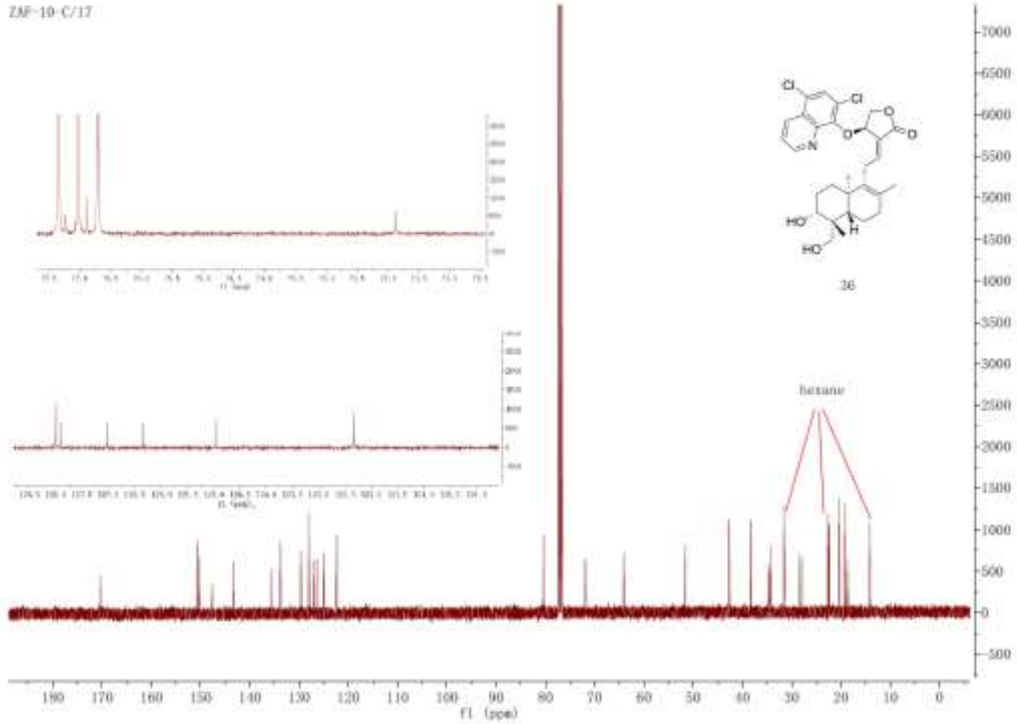

(14α)-(2’-Methyl-5’,7’-dichloro-8’-quinolinoxy)-9-dehydro-17-hydro andrographolide (**37**):

^{1}H NMR of **37**:

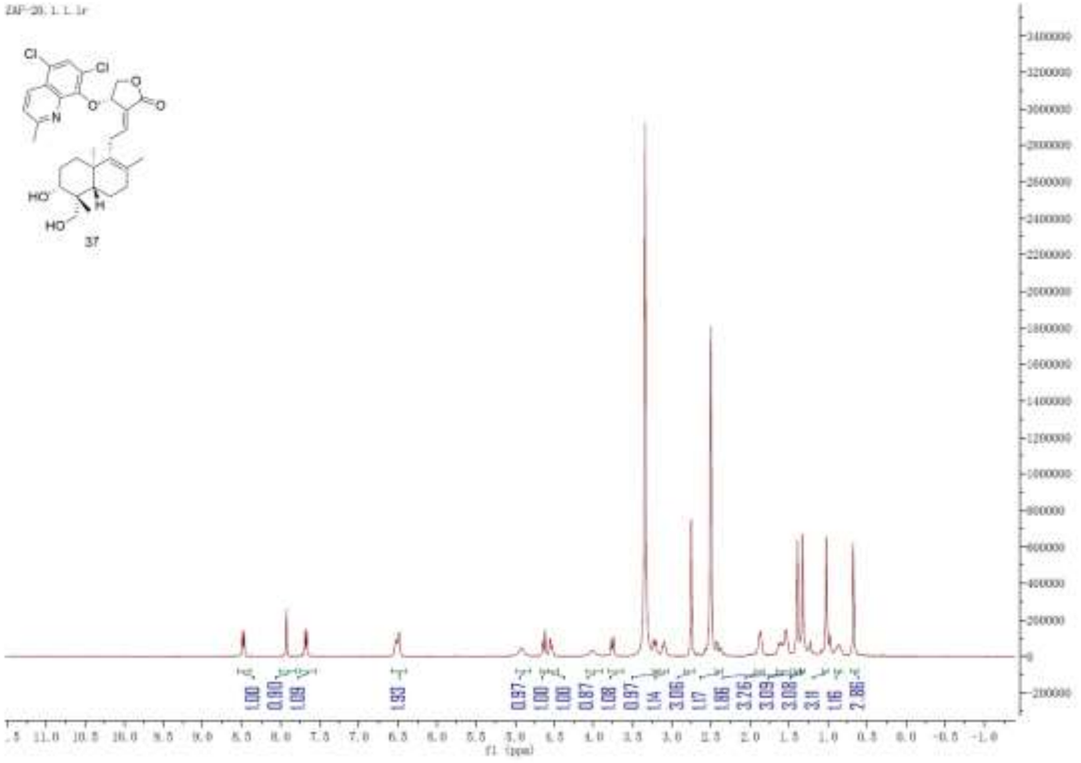

^{13}C NMR of **37**:

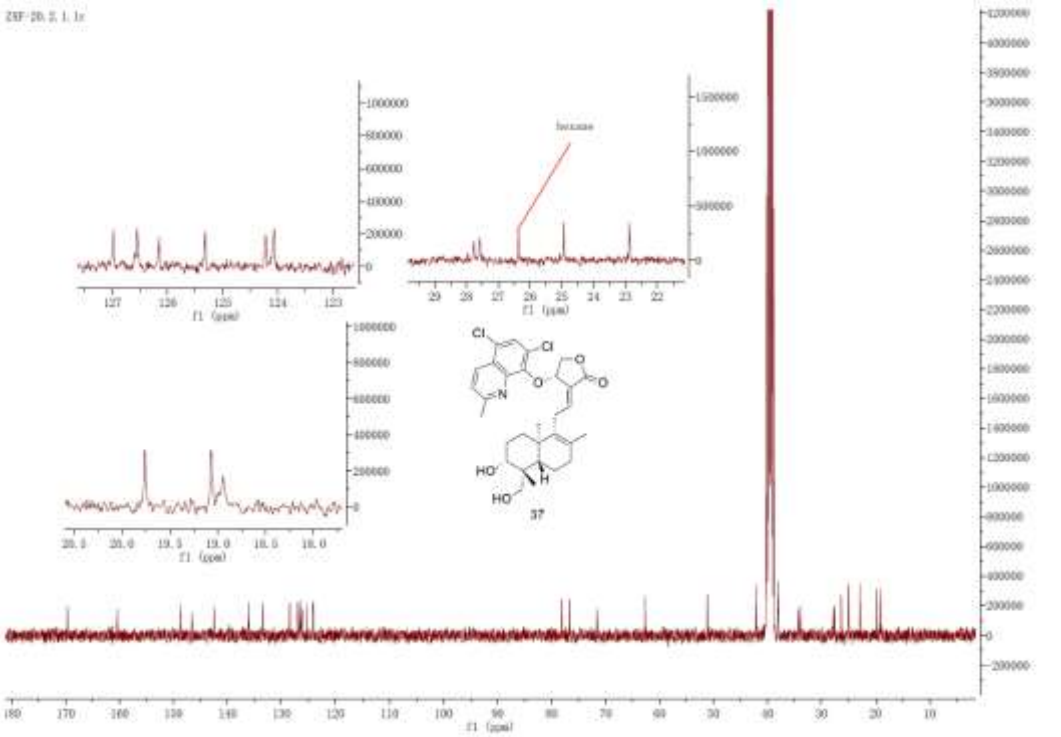

(14β)-(2’-Methyl-5’,7’-Dichloro-8’-quinolinoxy)-9-dehydro-17-hydro andrographolide (**38**):

^{1}H NMR of **38**:

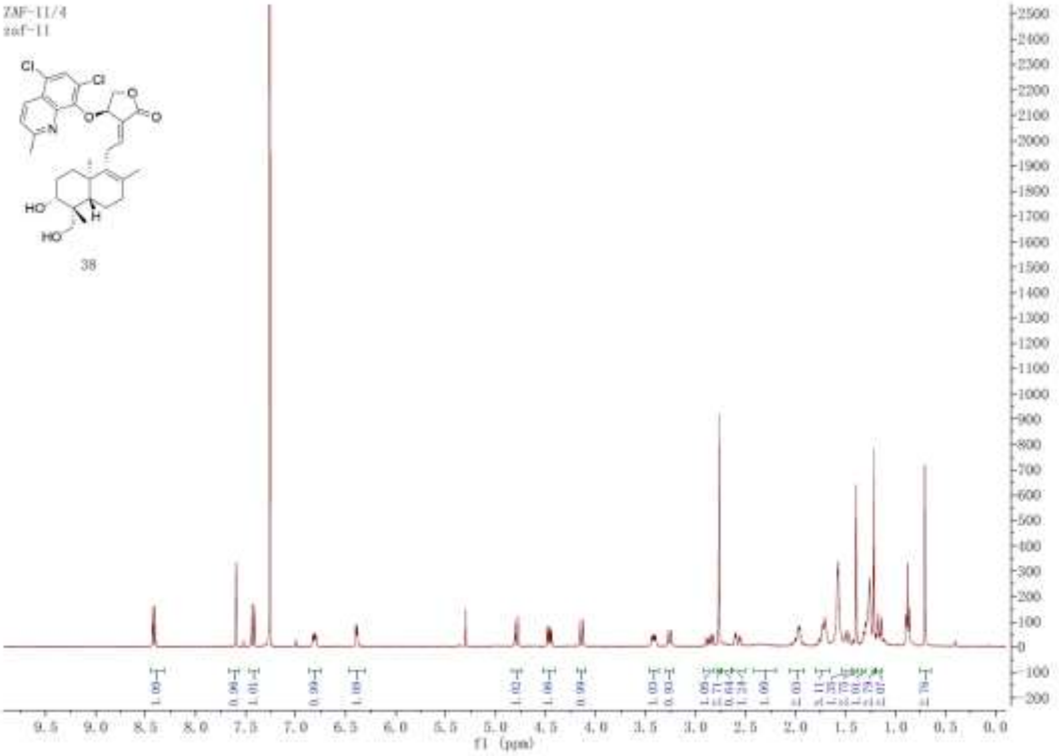

^{13}C NMR of **38**:

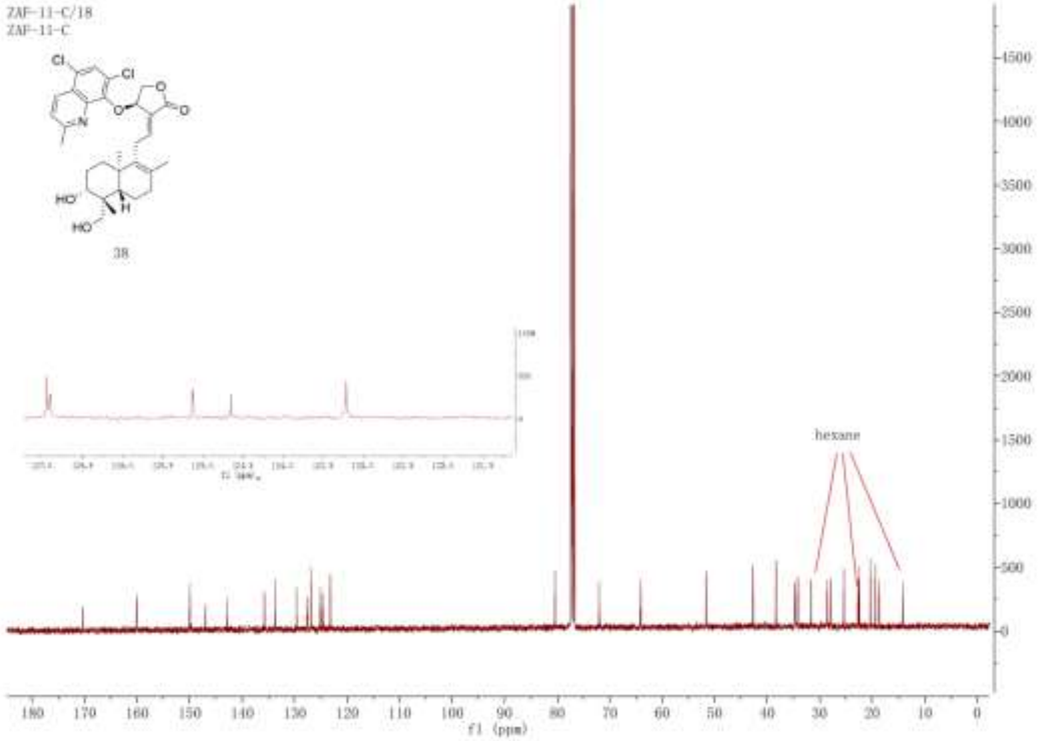

Supplement: Supplementary file 1 [file molecules-26-07660-s001.zip › Suplementary Materials-1492231.pdf]
